# Association of Shoulder Dysfunction with Mobility Limitation in Older Adults of the BLSA

**DOI:** 10.1093/geroni/igab046.3362

**Published:** 2021-12-17

**Authors:** Derik Davis, Kai Sun, Laurence Magder, Eleanor Simonsick

**Affiliations:** 1 University of Maryland School of Medicine, Ellicott City, Maryland, United States; 2 University of Maryland School of Medicine, Baltimore, Maryland, United States; 3 National Instute on Aging/NIH, Baltimore, Maryland, United States

## Abstract

Mobility limitation affects one-third of older adults; yet, the impact of shoulder dysfunction which effects roughly 20%, is inadequately documented. As arm swing is a fundamental component of ambulation, we investigated the cross-sectional association between shoulder range of motion (ROM) and walking endurance using time to walk 400m as quickly as possible and lower extremity performance using the expanded Short Physical Performance Battery (e-SPPB). Data are from 614 men (50.5%) and women aged ≥ 60 years (mean 71.8 ±8 years) in the Baltimore Longitudinal Study of Aging (BLSA) who performed bilateral shoulder elevation and/or bilateral shoulder external rotation (ER) during nurse-administered physical examination. We examined odds of poor 400m-walk and e-SPPB performance defined as the worst quartile associated with abnormal shoulder elevation (≤9%) relative to bilateral normal shoulder elevation and abnormal shoulder external rotation (≤5%) relative to bilateral normal shoulder external rotation (ER) in separate analyses. Analyses were adjusted for age, sex, weight and height. Adjusted odds (95% confidence interval) of poor 400m-walk performance associated with abnormal shoulder elevation (N=254) were 4.7 (1.1-19.5;p=0.035) and with abnormal shoulder ER (n=401) were 4.8 (1.4-16.7;p=0.010). Adjusted odds of poor e-SPPB performance associated with abnormal shoulder elevation (N=462) were 3.5 (1.6-7.7;p=0.002) and with abnormal shoulder ER (n=457) were 2.9 (1.1-7.4;p=0.030). Results suggest abnormal shoulder ROM is associated with poorer walking endurance capacity and lower-extremity functional performance in the relatively healthy older adults. Future research is warranted to develop novel screening paradigms that mitigate mobility limitation and functional decline in older adults with shoulder dysfunction.

